# *In Vivo* Evaluation of Cefuroxime Axetil-Loaded Bioadhesive Nanoparticles to Treat *Haemophilus influenzae-*Induced Otitis Media

**DOI:** 10.3389/fbioe.2022.884797

**Published:** 2022-04-29

**Authors:** Hong Lin, Yizhen Jia, Xiaohan Kong, Shiting Wang, Xiangyou Liu, Yang Liu, Yang Deng

**Affiliations:** School of Pharmaceutical Sciences (Shenzhen), Sun Yat-sen University, Guangzhou, China

**Keywords:** topical administration, drug delivery, bioadhesive nanoparticles, antibiotics, otitis media

## Abstract

Otitis media (OM) is a common disease in children. One of the most common pathogens causing OM is non-typeable *Haemophilus influenzae (NTHi)*. *NTHi* in the middle ear can be successfully eradicated by a regimen of oral antibiotics sustained for 7–10 days (e.g., cefuroxime axetil 250 mg/day for patients aged 3 months to 2 years and 500 mg/day for patients ages ≥2 years). However, lack of compliance is relevant to treatment failure or early relapse. In order to overcome these challenges, we have developed antibiotics-loaded bioadhesive nanoparticles (BNPs) that can adhere to the epidermis of the middle ear after local administration and significantly prolong the release time of antibiotics in the middle ear. Compared with oral administration of CA, local delivery of free antibiotic cefuroxime axetil (CA), and CA loaded non-bioadhesive nanoparticles (CA/NNPs), BNPs loaded with cefuroxime axetil (CA/BNPs) showed significantly longer retention time in the middle ear, resulting in continuous release of the drug and higher therapeutic efficacy against OM with only a single dosage. CA concentrations were maintained above the minimum inhibitory concentration (MIC) for *NTHi* throughout 7 days’ treatment. *NTHi* OM in a mouse model was successfully eradicated without causing tissue toxicity. CA/BNPs minimize systemic drug exposure through local administration, as demonstrated by undetectable levels in the blood.

## Introduction

Otitis media (OM) is one of the most common diseases in children, which is a host’s adaptive response to the bacterial invasion in the middle ear (Davidoss et al., 2018). Despite the availability of treatment guidelines, conjugate vaccines, and influenza vaccines, the incidence of otitis media remains high (Lieberthal et al., 2013). Acute OM affects 10% of people worldwide each year, and 51% of these cases occur in children under the age of 5 years (Monasta et al., 2012). Otitis media would lead to suppurative complications when it develops seriously, such as meningitis and mastoiditis (Klein 2000; Vergison et al., 2010). At the same time, the severity of otitis media will also significantly reduce the quality of patients’ lives. It may lead to OM children’s hearing impairment and backward development in cognition, language, social psychology, and other aspects, thus affecting their academic performance and interpersonal communication in the future (Boyle et al., 1994).

Over 70% of acute otitis media cases are caused by bacteria infections. Non-typeable *Haemophilus influenzae (NTHi)* is one of the major pathogens of acute otitis media, which may cause recurrent episodes and the failure of treatment (Haggard 2008; Leibovitz 2008; Welp and Bomberger 2020). Currently, there is no vaccine available for the treatment or prevention of diseases caused by *NTHi*. As a result, the present first-line medicine for *NTHi* infections is antibiotic treatment (Robledo-Avila et al., 2020). In order to eradicate otitis media caused by *NTHi*, it is vital to maintain an effective concentration of drugs in the middle ear throughout the treatment process. Thus, it requires a current treatment regimen that sticks with multiple doses of antibiotics daily for 7–10 days (Hoberman et al., 2016). The current clinical treatment standard for young children with otitis media is a 7- to 10-day course of oral broad-spectrum antibiotics. According to recent American Academy of Pediatrics guidelines, infants with OM should be treated with antibiotic therapy (Lieberthal et al., 2013). Thus, there are about 21% of all antibiotic prescriptions used in the United States for children, which represents OM as the primary reason for infancy’s systemic antibiotic exposure (Finkelstein et al., 2000).

Therapy is often terminated when clinical OM symptoms disappear. However, the pathogens in the middle ear may not be eradicated, which can result in recurrent OM and antibiotic resistance (Yang et al., 2018). The usage of oral broad-spectrum antibiotics may cause many adverse effects like rashes, vomiting, and diarrhea (Cunha 2001). According to recent research, the early exposure to systemic antibiotic is associated with health concerns (such as obesity and asthma) subsequently (Risnes et al., 2011; Cox and Blaser 2015). In addition, the systemic antibiotic abuse and incomplete compliance with the current multidose and multiday treatment regimens contribute to the continuous increase of antibiotic resistance, particularly in young children (Brook and Gober 2005; Zielnik-Jurkiewicz and Bielicka 2015).

In recent years, many different topical drug delivery systems have been developed to treat OM by avoiding system exposure. The encapsulation of ciprofloxacin into nanospanlastic vesicles enhanced noninvasive transtympanic delivery (Al-Mahallawi et al., 2017). Analogical results were acquired with levofloxacin encapsulated into polyethylene glycol 400 decorated nanoliposomes, and the drug deposition of this formulation inside the tympanic membrane was higher than that of free drug (Abdelbary et al., 2019). A thermosensitive *in situ* gelling hydrogel contained chemical permeation enhancers, and an antibiotic, allowing high drug concentration into the middle ear. However, it may immediately cause slight hearing defects after use (Yang et al., 2016).

**FIGURE 1:**
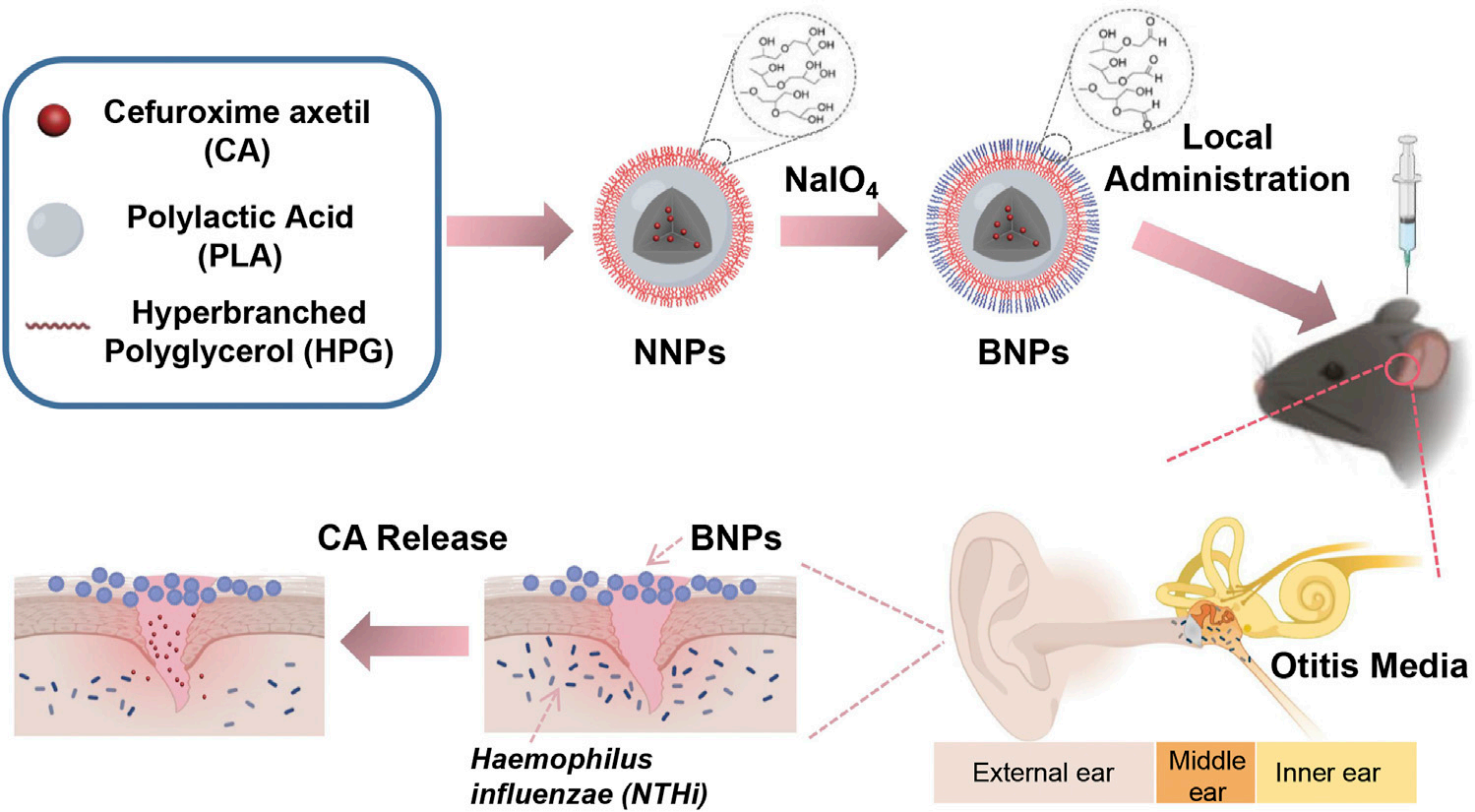
Nanoparticle design and therapeutic effects. Schematic showing the conversion process of NNPs into BNPs, in which vicinal diols on the surface of NPs were oxidized by sodium periodate into aldehydes. This process made BNPs have an additional bioadhesive. They adhered to the epithelial surface of the middle ear so that could prolong the retention exerting bacteriostatic effect efficiently.

Although the above treatment strategies have made great progress, the therapeutic effects can be further improved. In order to minimize the latent side effects and systemic exposure, it would be ideal to have effective care for the middle ear locally, which could treat OM effectively for its substantially high middle ear drug concentrations, instead of systemic administration. In this case, we have demonstrated the development of novel bio-adhesive nanoparticles (BNPs), which can prolong their retention on the epidermis of the middle ear adhesively after topical delivery (Figure 1). It can be prepared into nonadhesive nanoparticles (NNPs), which is “stealthy,” and can circulate for a long time through the veins after injection (Brook and Gober 2005). The vicinal diols on the surface coating of the NNPs can be converted to aldehydes when they are oxidized by sodium periodate for a short time. After that, it can form stable Schiff base interactions between aldehydes on the BNPs and amine groups of a protein, such as the lysine side chain or N-terminal (Deng et al., 2015; Deng et al., 2016). Our previous study has demonstrated that BNPs have been used successfully in reducing side effects of sunscreen (Deng et al., 2015), prolonging retention of elvitegravir intravaginally (Mohideen et al., 2017), and improving intravaginal therapeutic effects (Deng et al., 2016).

Herein, we describe the development of PLA-HPG-based BNP formulations encapsulating CA, a broad-spectrum antibiotic which was used to treat OM orally (Scott et al., 2001). The BNPs effectively eradicated acute OM caused by *NTHi* infection in a C57 mice model. This drug delivery system allows CA to prolong its release to the extent that it can last for at least 7 days in the middle ear to treat OM. Due to the adhesion design of drug delivery systems, a single dose of topical administration of the middle ear would cover an entire antibiotic course, which could improve treatment compliance and reduce side effects.

## Materials and Methods

### Materials

Cefuroxime axetil was provided by Meilun (Dalian, Liaoning, China). Polylactic acid (PLA) (Mw = 17 kDa) was obtained from SunLip Biotechnology (Shanghai, China). PLA-HPG was synthesized as previously described (Deng et al., 2014; Deng et al., 2015). Cyanine 5 (Cy5) and cyanine 7.5 (Cy7.5) were ordered from Aladdin (Shanghai, China). NaIO_4_, Na_2_SO_3_, anhydrous dichloromethane (DCM), sodium dodecyl sulfate (SDS), and HPLC-grade methanol were obtained from Macklin (Shanghai, China). N, N′-diisopropylcarbodiimide (DIC) was purchased from Sigma-Aldrich (St. Louis, MO, United States). Potassium dihydrogen phosphate and potassium ammonium dihydrogen phosphate were chemical reagents (Guangzhou, Guangdong, China).

### PLA-Cy5 Synthesis

The synthesis of PLA-Cy5 was formed from the reaction between the amine group from Cy5-NH_2_ and the carboxylic acid group from PLA; 1.95 g PLA was dissolved in DCM, and then 15 mg Cy5-NH_2_ and 0.02 ml DIC were added into the solution. The reaction was stirred for 1 day at room temperature. After the addition of cold diethyl ether, the product precipitated and was collected by centrifugation (4,500 rpm, 15 min), which was dried under vacuum for 2 days. The synthesis of PLA-Cy7.5 was consistent with PLA-Cy5.

### Nanoparticle Preparation

To prepare NNPs, 60 mg of PLA-HPG and 120 mg of PLA were dissolved in 2 ml of DCM. For dye-loaded nanoparticles, either PLA-Cy7.5 or PLA-Cy5 was dissolved in 2 ml of DCM. For drug-loaded nanoparticles, CA was dissolved in 2 ml of DCM. The drug or dye solution was then combined with the polymer solution, resulting in a polymer/dye or polymer/drug solvent mixture. The resulting solution was added to 8 ml deionized (DI) water under vortex and sonicated with a probe sonicator (3 × 10 s each). Then, the emulsion was diluted in 20 ml of DI water and subsequently placed on a rotavapor for 30 min. The particle solution was washed and centrifuged 3 times with DI water (4,500 rpm, 15 min) before being suspended in DI water. NNPs were stored at −20°C until use.

To convert NNPs into BNPs, NNPs were incubated with 0.1 M NaIO_4_ (1:1 volume ratio) for 2 min, and then the reaction was quenched with 0.2 M Na_2_SO_3_ (1:3 volume ratio). BNPs were washed and centrifuged 3 times with DI water (4,500 rpm, 15 min), and finally, the precipitate was resuspended in DI water.

### *In Vitro* Characterization and Drug Release of Nanoparticles

The laser Doppler electrophoresis and dynamic light scattering of a Zetasizer Nano ZS (Malvern, United Kingdom) were used to determine the surface charge, diameter, and polydispersity index (PDI) of nanoparticles (*n* = 5). Transmission electron microscopy (TEM) (JEM-1400 electron microscope (JEOL Ltd., Japan)) was used to characterize the morphologies of nanoparticles. The concentration of Cy5 (em/ex 670/650 nm) and Cy7.5 (em/ex 808/788 nm) was quantified by IVIS Lumina XR Series Ⅲ (Perkinelmer, United States). Dye loading was determined from a standard curve. CA loading efficiency in nanoparticles was determined by high-performance liquid chromatography (HPLC) (LC-20A, Shimadzu, Japan) with C18 analytical column (120-5-C18 EPS, Ecosil, Germany). NPs were diluted 10-fold in methanol and filtered with a 13-mm HPLC syringe filter. Methanol/water supplemented with 0.2 M ammonium dihydrogen phosphate was used as the mobile phase, and the wavelength of the UV detector was set at 278 nm. The drug loading efficiency was calculated from a standard curve.

*In vitro* drug release of NNPs and BNPs was determined by shaking a 500 µL suspension of free CA, CA-loaded NNPs, or BNPs (*n* = 3) 40 fold in PBS supplemented with 1% SDS in a shaker (Bluepard, China) at 37°C. The resulting solution was centrifuged in millipore tubes (100 kDa MWCO (molecular weight cutoff), 7,000 rpm 20 min). At each time point, millipore tubes (*n* = 3) were removed, and the remaining CA level in each tube was quantified with HPLC as described previously. At each time point, the CA solution in the centrifuge tubes was replaced with fresh buffer.

*In vitro* PLA-Cy5 release of Cy5-NNPs and Cy5-BNPs was determined by shaking a 500 µL suspension of Cy5-NNPs and Cy5-BNPs (*n* = 3) 40 fold in PBS supplemented with 1% SDS in a shaker at 37°C. The resulting solution was centrifuged in millipore tubes (100 kDa MWCO (molecular weight cutoff), 7,000 rpm 20 min). At each time point, millipore tubes (*n* = 3) were removed, and the remaining PLA-Cy5 level in each tube was quantified with a microplate reader (BioTeck, United States).

### Cytotoxicity Analysis

To evaluate the influence of NNPs and BNPs on cell viability, human dermal fibroblasts (hFbs, gifted from the First Affiliated Hospital, Sun Yat-sen University, China) were cultured in Dulbecco’s modified eagle medium (DMEM) supplemented with 10% fetal bovine serum and 1% penicillin/streptomycin. hFbs in 100 µL of DMEM were plated in each well of a 96-well plate at a density of 3,000 cells per well and maintained at 37°C in the presence of 5% CO_2_ to be incubated overnight. The medium was removed on the second day, and then 100 µL of DMEM, blank NNPs, blank BNPs, CA/NNPs, or CA/BNP DMEM suspension was added to cells. Cells were incubated at 37°C for 24 h, and then cell viability was quantified with Cell Counting Kit-8 using a microplate reader.

To evaluate the influence of free CA on cell viability, 0.1, 0.5, 1, and 10 mg/ml free CA were used to treat human dermal fibroblasts. After incubation at 37°C for 24 h, the cell viability was quantified with Cell Counting Kit-8 using a microplate reader.

### *In Vivo* Nanoparticle Retention in the Middle Ear

Animal experiments were reviewed and approved by the Administrative Committee of Animal Research at the Sun Yat-Sen University; 5 µL of either Cy7.5-NNPs or Cy7.5-BNPs were injected into the right middle ears of mice (6- to 8-week-old C57Bl/6J female) (*n* = 5). At each time point (5 min, 1 day, and 2, 3, 4, 5, 6, and 7 days), the right ear of mice was imaged using IVIS, and then we used the software to quantify the signals in the middle ear of mice. The tissue autofluorescence from untreated controls was eliminated by adjusting the lower end of the fluorescence intensity range. The treated ways for each group of animals were the same. The imaging methods were described previously (Mohideen et al., 2017).

To further evaluate the nanoparticle retention of BNPs, mice (*n* = 3) were treated with 5 μL of Cy5-NNPs or Cy5-BNPs. Mice were sacrificed at each time point (5 min, 1 day, and 2, 3, 4, 5, 6, and 7 days) after treatment, and the middle ear tissue was excised. The collected middle ears were imaged using a live imaging instrument.

### Retention Profile of Cefuroxime Axetil in the Middle Ear

A total of 5 μL of CA/NNPs or CA/BNPs were applied to every right middle ear. At each time point (5 min, 1 day, and 2, 3, 4, 5, 6, and 7 days), mice (*n* = 3) were sacrificed, and the middle ear tissue was excised. The middle ear tissues were homogenized in 1 ml of methanol. After homogenization, samples were centrifuged at 10,000 rpm for 10 min at 4°C, and the supernatant was collected and evaporated for 24 h. Samples were then reconstituted in 0.4 ml of methanol and filtered. CA in the processed tissue samples was quantified using HPLC.

### *NTHi* Otitis Media Mouse Model

Mice were operated on to occlude the transcervical eustachian tube (Khomtchouk et al., 2020). In short, we used a transcervical approach to identify the bony eustachian tube, and then verified medial to the tympanic bulla. After that, a piece of surgical thread was used to ligate the transcervical eustachian tube. Mice recovered by 24 h before inoculation of the middle ear. Isolates of *NTHi* grown to the mid-log phase were diluted in phosphate-buffered saline (PBS), and about 10,000 CFUs in 5 μL were introduced directly into each middle ear bullae under aseptic conditions (Hirano et al., 2016). Daily otomicroscopy was performed to monitor the presence of fluid in the auditory bullae, and signs of infection, including bulging tympanic membrane (TM), redness, and swelling, and pictures, were noted. Three days after *NTHi* infection, TMs of the animals to receive the formulation were observed with the speculum of an otoscope, after which 5 µL saline, blank NNPs, blank BNPs, CA/NNPs, CA/BNPs, and free CA were administrated into the middle ear through intratympanic injection by the sterile syringe. The final group received an equivalent CA orally. The concentrations of free CA, oral CA, and CA in the CA-loaded particles were 0.5 mg/ml. The middle ear was washed daily with 10 μL physiologic saline, and the cleanout fluid was centrifuged (10 min, 3,000 rpm, 4°C). Removing the supernatant and using 100 μL PBS to resuspend the precipitate, three serial 10-fold dilutions were prepared; 10 μL of each dilution was immediately plated onto a blood agar plate. Otoscopy and bacterial culture count checks were carried out every 1–2 days until the bacteria culture count was zero. Serial animal serum samples were obtained at 0, 2, and 4 days to determine systemic cefuroxime levels by HPLC.

### Histopathology

Nanoparticles were administered to the middle ears of live healthy or OM mice. Seven days later, they were accepted for euthanasia. The ears were excised, and the major organs (heart, liver, spleen, lung, kidney, and brain) of each group were isolated and immediately fixed in 4% paraformaldehyde overnight, then decalcified, embedded in paraffin, sectioned, and stained with H&E by the Servicebio Company (Guangzhou, China; fee for service), using standard techniques. All stained paraffin sections were evaluated under light microscopy.

### Statistical Analysis

All statistics were processed using GraphPad Prism software. The error bars in each figure had been indicated properly, which were presented as the mean ± standard deviation (SD). Statistical analysis was conducted using a paired or unpaired Student’s t-test with GraphPad Prism software. Significance was represented on plots as ****, *p* < 0.0001; ***, *p* < 0.001; **, *p* < 0.01; *, *p* < 0.05; ns, *p* > 0.05.

## Results

### Nanoparticle Synthesis and Characterization

**FIGURE 2:**
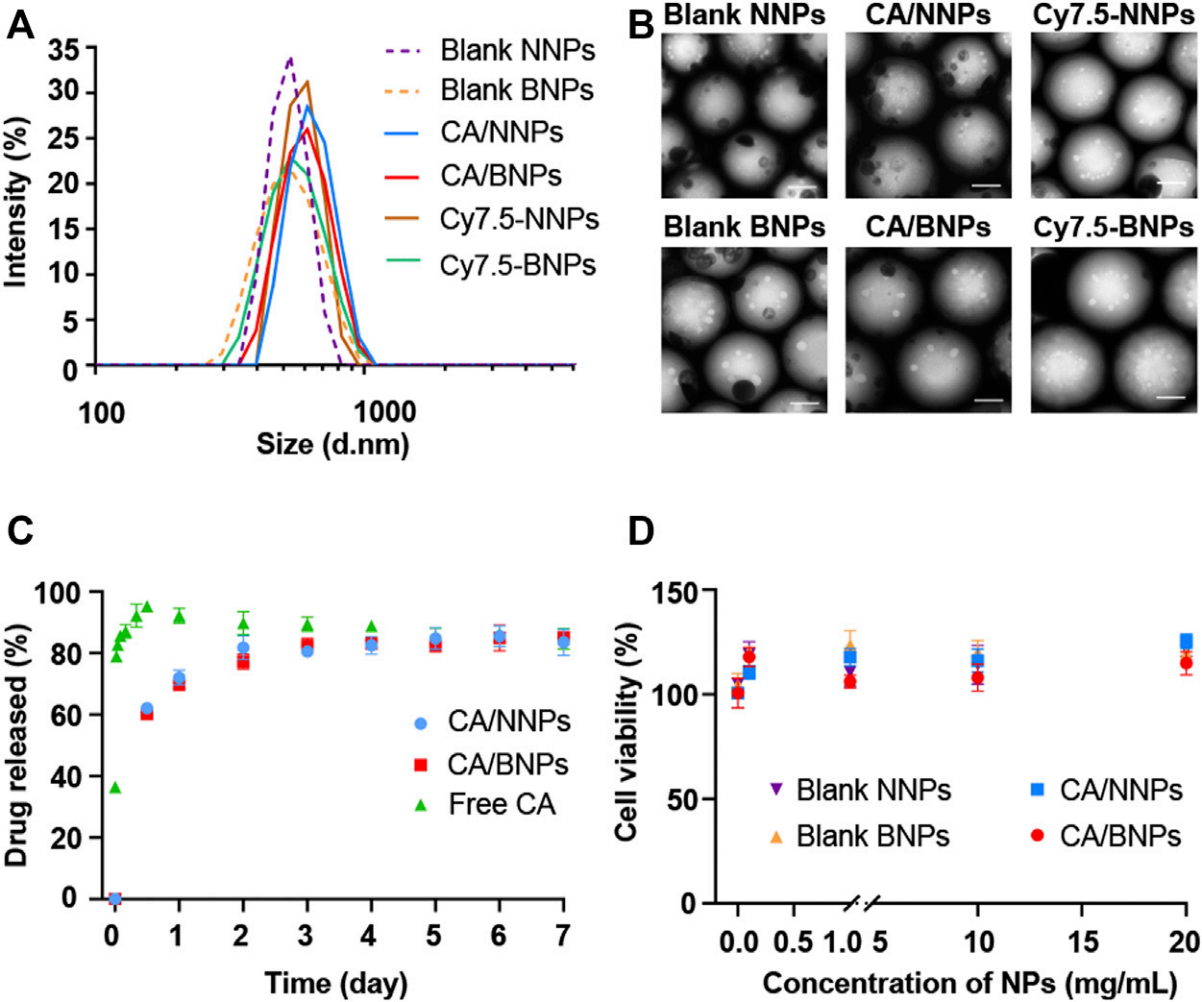
*In vitro* nanoparticle characterization. **(A)** Hydrodynamic diameters of nanoparticle formulations. Data are means ± SD (*n* = 3). **(B)** TEM images of blank NNPs, blank BNPs, CA/NNPs, CA/BNPs, Cy7.5-NNPs, and Cy7.5-NNPs (scale bars: 200 nm). **(C)** *In vitro* release profile of free CA, CA/NNPs, and CA/BNPs. Data are means ± SD (*n* = 3). **(D)** Cell viability of hFbs incubating with blank NNPs, blank BNPs, CA/NNPs, and CA/BNPs for 24 h *in vitro*. Data are means ± SD (*n* = 4).

The HPG hydrophilic shell and PLA hydrophobic core could be formed after PLA-HPG self-assembling in aqueous solution, as PLA-HPG was an amphiphilic copolymer. As a result, hydrophobic polymer PLA and hydrophobic small-molecule drug CA could be incorporated into the hydrophobic core during the PLA-HPG self-assembling process. In order to seek the optimal proportion of synthetic materials, several different ratios were tried. Based on the sizes of nanoparticles and CA encapsulation efficiency, the optimal formulation was PLA-HPG concentration of 10 mg/ml, PLA concentration of 20 mg/ml, and 3.3% (w/w) of CA. The hydrodynamic sizes of blank NNPs, blank BNPs, CA/NNPs, CA/BNPs, Cy7.5-NNPs, and Cy7.5-NNPs were 568.4, 584.8, 609.9, 610.5, 606.2, and 601.8 nm (Figure 2A). The polydispersity index of CA/NNPs and CA/BNPs was lower than 0.3, which was also confirmed by the result of TEM. Through the TEM results, the morphology of nanoparticles was spherical, and the particle size of each particle was relatively uniform. The sizes of blank NNPs, blank BNPs, CA/NNPs, CA/BNPs, Cy7.5-NNPs, and Cy7.5-NNPs were 453.6, 473.6, 473.3, 560.5, 464.4, and 557.9 nm, respectively, which were smaller than hydrodynamic sizes. This is mainly due to the different preparation states of the samples. The dried state of samples was for TEM imaging and the hydrated state of samples was for dynamic light scattering. The latter showed larger hydrodynamic volume because of the solvent effect in the hydrated state (She et al., 2013) (Figure 2B).

The CA encapsulation efficiency of BNPs and NNPs was about 16.17%. As shown in Figure 2C, free CA was rapidly released to 95% within 12 h, which was near full release. About 69% of the cefuroxime axetil in CA/NNPs and CA/BNPs was released during the initial 24 h of incubation when they were incubated in 1% SDS at 37°C. Compared to free CA, CA/BNPs and CA/BNPs could release CA for at least 7 days, and this shows that they could be used as a drug delivery system for continuous drug release (Figure 2C). The stability of nanoparticles was verified through *in vitro* PLA-Cy5 release from Cy5-NNPs and Cy5-BNPs. There was no PLA-Cy5 detected during 7 days of release experiment. Therefore, NNPs and BNPs could be stable in the aqueous phase for at least 7 days. In order to test NP toxicity, hFb cells were treated with CA/NNPs and CA/BNPs at different concentrations at the very start. Cells were assessed for their viability after 24 h. There was no decrease in the cell viability with the addition of NNPs and BNPs, compared with untreated control in hFbs at all concentration tests, which ranged from 0.1 mg/ml to 20 mg/ml. This result demonstrated that the NNPs/BNPs were suitable as nanodrug carriers, as they display low levels of cytotoxicity (Figure 2D). The cell viability was above 95% with the addition of free CA, ranged from 0.1 mg/ml to 1 mg/ml, which demonstrated 0.5 mg/ml free CA that was administrated *in vivo*, and exerted almost no toxicity on cells or tissues. In addition, CA was one of the systemic antibiotics approved by the Food and Drug Administration for the treatment of acute OM (Erramouspe and Heyneman 2000).

### Bioadhesive Nanoparticles Enhance Retention of Nanoparticles in the Middle Ear

**FIGURE 3:**
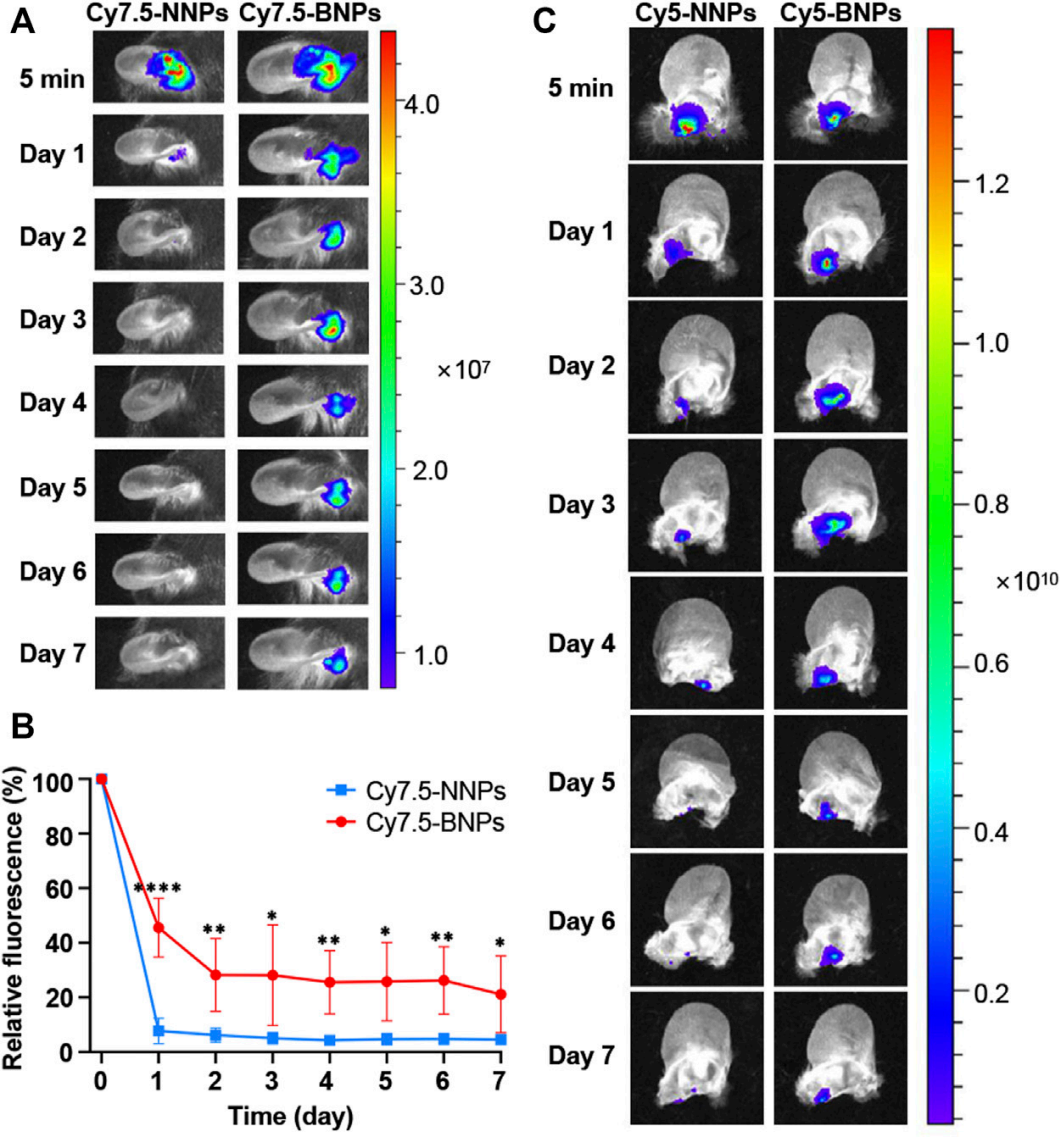
Retention of nanoparticles in the middle ear. **(A)** The retention of the Cy7.5-NNPs and Cy7.5-BNPs monitored with NIR imaging after administration *in vivo*. Scale bar represents relative dye intensity. **(B)** Quantification of fluorescence retained in the middle ear for 7 days. Data are means ± SD (*n* = 4). *p < 0.05, **p < 0.01, ***p < 0.001, ****p < 0.001, compared with Cy7.5-NNP group (Student’s t-test). **(C)** Living imaging of harvested middle ear tissue treated with either Cy5-NNPs (left panel) or Cy5-BNPs (right panel) for 7 days.

In order to determine whether BNPs had the advantage in prolonging retention in the middle ear compared to NNPs, PLA-Cy7.5 contained NNPs or BNPs were injected into the middle ears of normal mice. The model’s middle ear was washed daily with saline, and the remaining dye percentage was quantified in 0–7 days. The usage of dyes for the retention of nanoparticles had been documented previously for the test of prolonging the retention in vaginal tissue (Mohideen et al., 2017). Thus, it is reliable for the fluorescence intensity of the dye to serve as the marker to represent corresponding nanoparticles. After the administration of Cy7.5-NNPs or Cy7.5-BNPs, their fluorescent signals in the middle ear were relatively similar, which were strong initially (5 min after administration) (Figure 3A). Furthermore, 45.5% Cy7.5-BNPs remained in the middle ear over 24 h, compared to Cy7.5-NNPs which only remained at 7.7% (Figure 3B). This suggested that Cy7.5-NNPs may have been washed out during the cleaning of the middle ear, while Cy7.5-BNPs could stagnate because of their preferable adhesivity. The fluorescent signal of Cy7.5-NNPs was relatively feeble by the end of the 7 days. In contrast, Cy7.5-BNPs were observed to have 21.1% retention after 7 days post-treatment, which was nearly 4-fold higher than that of Cy7.5-NNPs. These differences between BNPs and NNPs in adhesion properties were found to be highly significant. Therefore, BNPs can be a stable depot system to prolong the duration of CA.

To further verify the retention of NNPs and BNPs in the middle ear *in vivo*, another dye-conjugate, PLA-Cy5 was mixed into NNPs and BNPs, which were injected into the middle ears of mice. The ears of animals were harvested, at each time point (5 min, 1 day, and 2, 3, 4, 5, 6 and 7 days), and the remaining staining was photographed and measured after the unfolding of the ear. Similar to the results of *in vivo* photographing, Cy5-NNPs decreased rapidly after the first day and nearly disappeared on the seventh day, while Cy5-BNPs remained strong, and fluorescent intensity lasted up to 7 days, which confirmed the results of *in vivo* photographing (Figure 3C). The oxidized converts of vicinal diol to aldehydes made BNPs adhesive, which caused further retention of nanoparticles in the middle ear through binding to the surface protein.

### Cefuroxime Axetil/Bioadhesive Nanoparticles Enhance the Duration of Cefuroxime Axetil in the Middle Ear

**FIGURE 4:**
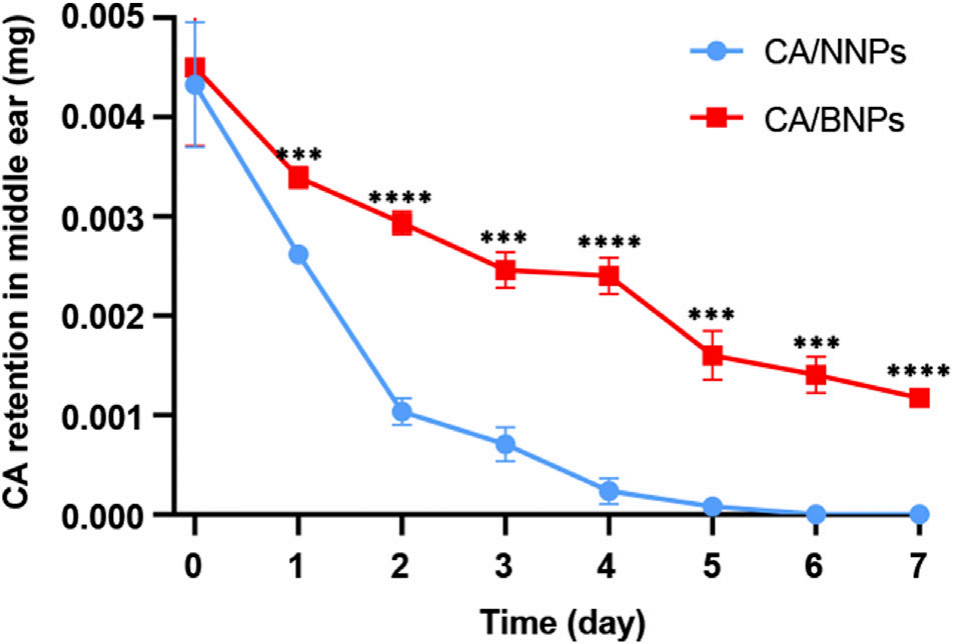
Quantification of CA retained in the middle ear for 7 days. Data are means ± SD (*n* = 3). ***p < 0.001, ****p < 0.001, compared with the CA/NNP-treated group (Student’s t-test).

In order to confirm the CA/BNPs can prolong the residence time in the middle ear, CA/NNPs and CA/BNPs were injected into the middle ears of the OM mouse model, and the middle ears were collected at 5 min, first day, and second, third, fourth, fifth, sixth, and seventh days after treatment. Followed by homogenate and extraction, the concentration of CA in the supernatant was determined by HPLC. As was shown, CA retention decreased over time, which was probably because CA released from the nanoparticles was washed out with the lavage fluid or metabolized by middle ear tissues. On the seventh day, the amount of CA in the CA/BNP-treated group was 0.0012 mg. Meanwhile, when calculating with a dosage volume of 5 μL, the concentration of free CA in CA/BNP groups was about 6 mg/L on the seventh day according to the *in vitro* drug release profile, which was higher than the MIC concentration of 0.12–4 mg/L (Harrison et al., 2009), whereas CA of the CA/NNP-treated groups was almost nonexistent (Figure 4). This suggested that CA/BNPs had a sustained bacteriostatic effect in the middle ear and reduced the possibility of bacterial recurrence.

### Bioadhesive Nanoparticle Delivery System Cured Otitis Media *In Vivo* in the Otitis Media Mouse Model

**FIGURE 5:**
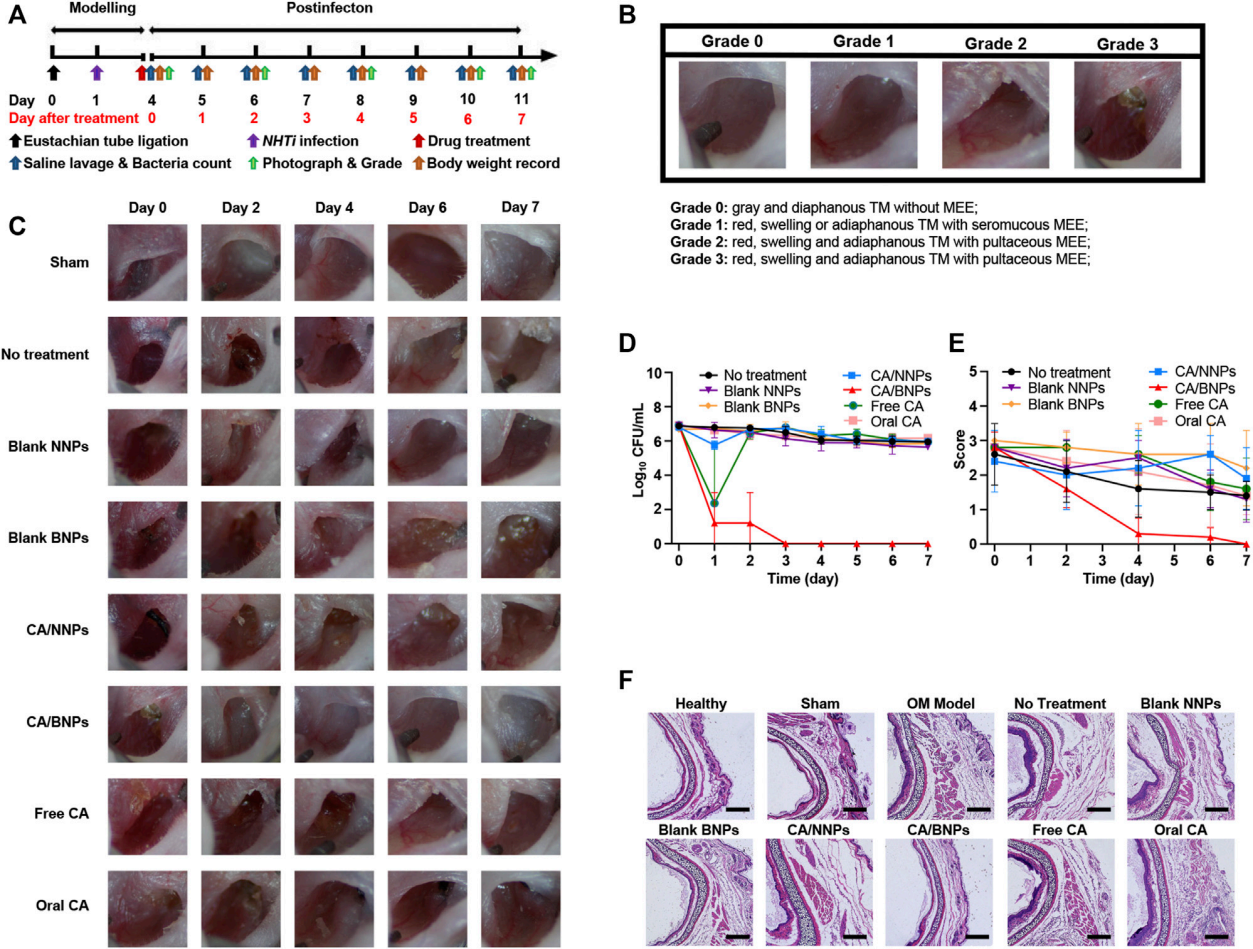
*In vivo* therapeutic efficacy of CA/BNPs on OM mice. **(A)** Generation of a *NHTi* suppurative otitis media mouse model. (model development scheme). The mouse underwent surgery of eustachian tube ligation 1 day before *NHTi* inoculation. CA/NNPs or CA/BNPs were administrated at day 4 after infection for 3 days. After treatment, the mice were lavaged with physiological saline and monitored *via* bacterial counting every day. **(B)** Grading schematic for the establishment of OM in mice. **(C)** The photographs of the middle ear in different groups on days 0, 2, 4, 6, and 7 could observe the severity degree of middle ear visually. **(D)** Time course of bacterial CFU from middle ear irrigation solution from mouse with OM from *NTHi* treated with saline, blank NNPs, blank BNPs, CA/NNPs, CA/BNPs, free CA, or oral CA. Data are means ± SD (*n* = 5). **(E)** The score of the middle ear. Data are means ± SD (*n* = 5). **(F)** H&E-stained cross sections of the middle ear after different treatment for 7 days. Scale bar: 200 nm (magnification = ×10). These images are representative of the middle ear sections from *n* = 5 mice for each group.

*NTHi*-induced OM was established in C57 mice as the time line of the procedure in Figure 5A. In short, *NTHi* was inoculated into the middle ear of the animals 24 h later, after the ligation of the eustachian tube with surgical thread; 3 days after inoculation, the middle ear of the animal was investigated and pictured through a stereo microscope, the next was to grade the severity of the infection. Score grade three indicated the successful establishment of the infection, which would trigger nanoparticles’ treatments described on that following day. The infected animals were injected with 5 µL test formulations directly into the middle ear. Afterward, the middle ear was washed with physiological saline every day. The irrigation solution was cultured for the bacterial count determination in the middle ear. The bacterial count in the middle ear declined by 99.9% and was perceived as an indication of healing.

According to the severity of the acute OM, the characteristics of middle ear effusion (MEE) were recorded once in 2 days and graded by the degree order of inflammation. The criteria were referenced and modified by Giebink and Wright (1983). As is shown in the graph (Figure 5B), the following were observed: 0, gray and diaphanous TM without MEE (healthy); 1, red, swelling, or adiaphanous TM with seromucous MEE; 2, red, swelling, and adiaphanous TM with seromucous MEE; and 3, red, swelling, and adiaphanous TM with pultaceous MEE. The experimental animals were divided into eight groups: sham operation group, no treatment, CA/BNPs, CA/NNPs, blank BNPs, blank NNPs, free CA, and oral CA. After the first day, the CA/BNP group had obvious signs of cure, while the middle ear in other groups remained red, swollen, and had adiaphanous TM with pultaceous MEE (Figure 5C). Symptoms including inflammation and bulging of the TM resolved within 24 h after treatment with CA/BNP began.

The time course of the number of *NTHi* colonies (CFU/ml) in the saline lavage fluid in each group was provided in Figure 5D. After 7 days of treatment, although other groups existed with slight cure, the CA/BNPs were completely cured and the scores of this group were 0 as the TM was gray and diaphanous without MEE (Figure 5E). This was again confirmed by biopsy of the eustachian tube, and it was seen that the thickness of the tube cross section in the CA/BNP group was the same as that in the healthy group. In addition, it was evident that the thickness of the tube cross-section in the sham operation group, no treatment, and CA/NNP, blank BNP, blank NNP, free CA, and oral CA groups was much thicker than that in the healthy group due to eustachian tube ligation and inflammation after bacterial inoculation (Figure 5F).

### *In Vivo* Toxicity Evaluation of Nanoparticles

**FIGURE 6:**
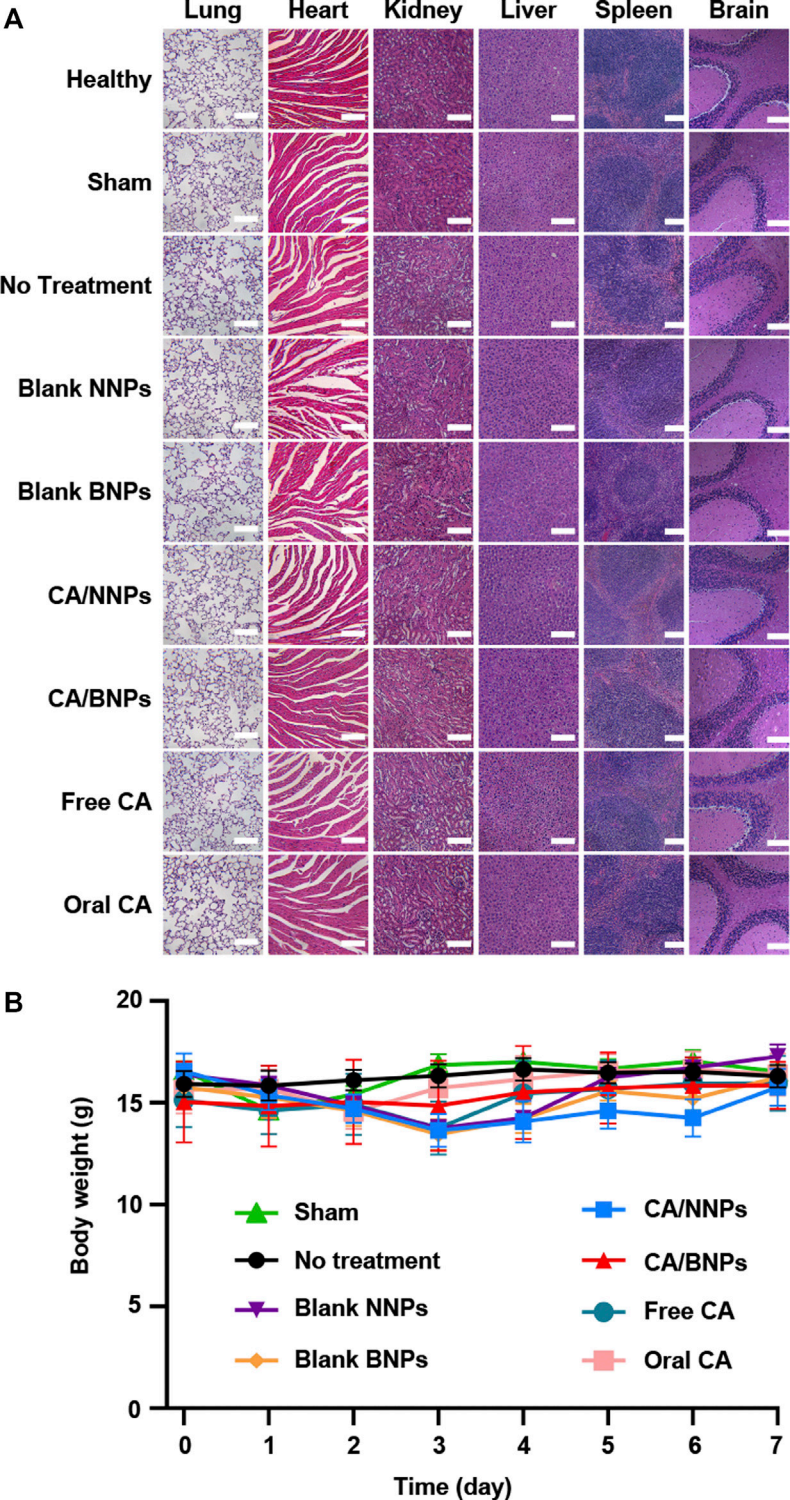
*In vivo* toxicity evaluation. **(A)** H&E staining of the major organs of the mice 7 days after different treatments (scale bar = 500 nm). **(B)** Body weight changes of mice in different groups.

*In vivo* toxicity of CA/NNPs and CA/BNPs was analyzed by H&E stained sections of various organ tissues (Figure 6A). The H&E stained sections of various organs in healthy mice and model mice treated with CA/NNPs and CA/BNPs displayed similar morphology. Therefore, there was no observed biological toxicity or inflammation in any group, as assessed by the H&E stained sections of the heart, liver, spleen, lung, kidney, and brain. In addition, the body weights of the mice were recorded every day, and it showed no severe body weight loss in the CA/BNP-treated group (Figure 6B). A significant potential advantage of single-dose injection delivery of drug was that it could achieve high topical antibiotic levels in the middle ear with the minimal systemic distribution. To determine the CA level in the serum of the mice, the blood samples were collected at set intervals from the submandibular space of the mice treated with CA/BNPs. CA was not detected in serum at 0, 2, or 4 days by HPLC. As it was hard for CA to be absorbed into the blood through the middle ear local administration in the first place, it was more difficult for CA to distribute to other organs through blood circulation. The level of CA was high in the middle ear, and no CA was detected in the serum, which had provided strong evidence for the low or no systemic exposure to antibiotics.

## Discussion

Oral administration of antibiotics and surgery are most commonly used in the clinical treatment of otitis media at present. However, oral administration of CA may cause mild gastrointestinal events (e.g., nausea and diarrhea) in infants and young children (File et al., 1997), while surgery is thought to be invasive. A hydrogel containing an antibiotic and CPEs had also been studied which would immediately cause slight hearing defects after usage (a few minutes) (Yang et al., 2016). In addition, compared to gel formulation, the nanoparticle solution was fluid, which could be more evenly distributed on the surface of the infected middle ear. However, there is limited research on nanoparticles of OM because most nanoparticles have no adhesion property to prolong their retention in the middle ear with the irrigation of the middle ear during the treatment. Here, we demonstrate the development of a potentially long-acting bioadhesive nanoparticle which can extend the retention of nanoparticles and the release of antibiotics more than 7 days. CA/NNPs and CA/BNPs were prepared for the suitable sizes of about 600 nm, and they were also optimized to provide a controlled dissolution curve of drug release *in vitro*. The release profile of CA/BNPs and CA/NNPs were similar to those of nanoparticles prepared by PLA-HPG as reported previously. In addition, the cytotoxicity test also showed that the nanoparticles were safe to use.

After sodium periodate (NaIO_4_) treatment, the vicinal diols on the surface of NNPs were oxidized to aldehyde groups, which was validated by H^1^ NMR and Schiff’s agent analysis previously (Deng et al., 2015). The aldehyde groups and the amino groups on the surface of the protein produce the Schiff-base bond, which would make BNPs bioadhesive (Deng et al., 2015; Deng et al., 2016). Although it is known that they have low toxicity (O'Brien et al., 2005), their distribution is limited by the covalent binding of aldehydes to BNPs on the BNP surface. In addition, it is common to find aldehydes in many foods and metabolites, and they are easily detoxed by aldehyde dehydrogenase (Vasiliou et al., 2013), which accounts for the low toxicity of BNPs. Drug-loaded BNPs adhered to proteins on the epidermis of the middle ear, thus prolonging the retention time of nanoparticles in the middle ear. To verify this hypothesis, we used Cy7.5-loaded NNPs and BNPs as tracers that were similar in size to CA/NNPs and CA/BNPs, and they had substantial retention in the middle ear for at least 7 days. Compared with conventional particles such as Cy7.5-NNPs, BNP particle retention is significantly enhanced. Even after 7 days of topical administration, about 21 percent of Cy7.5-BNPs remained in the middle ear, which may have a long-term therapeutic effect on otitis media disease and also reduce the probability of recurrence.

The platform described above can be applied for the treatment of acute OM topically with a single dose. In model mice treated with CABNPs, the concentration of CA in the middle ear was maintained above the MIC of *NTHi* throughout the 7-day treatment. The persistent high concentration of CA indicated that this formulation was effective in treating OM, eventually eliminating inflammation, eradicating the infection, and returning the middle ear to normal. Although local concentration was high, no drug was detected in the serum, which was potentially important for avoiding systemic toxicity that leads to the emergence of resistant bacteria. However, the overuse of systemic antibiotics to treat OM was thought to be partly responsible for increasing resistance to nasopharyngeal pathogens (Barkai et al., 2009). On the other hand, receiving oral therapy in the pediatric population may lead to low compliance because of the relatively bitter taste of cefuroxime axetil suspension (Steele et al., 2001). Therefore, the preparation of a bioadhesive nanoagent of cefuroxime axetil can improve patient compliance with a single dose. Simultaneously, the composition of CA/BNP prescription is simple, which is relatively easy for clinical transformation.

We have developed a single-dose, topical antibiotic treatment regimen as well as demonstrated its effectiveness in animal models. The safety concerns of the nanoscaled drug delivery system could be omitted because of its local bioadhesiveness and confinement. Yet the safety of the overall formulation may need to be validated in follow-up preclinical studies and clinical trials before the developed potent therapeutic NPs could be applied clinically. It is promising to develop the designed nanopreparation for clinical application due to the optimized and simplified drug delivery carrier and the well-proven therapeutic effect.

## Data Availability

The raw data supporting the conclusions of this article will be made available by the authors, without undue reservation.
